# Too Much of a Good Thing? A Governing Knowledge Commons Review of Abundance in Context

**DOI:** 10.3389/frma.2022.959505

**Published:** 2022-07-13

**Authors:** Michael J. Madison, Brett M. Frischmann, Madelyn R. Sanfilippo, Katherine J. Strandburg

**Affiliations:** ^1^School of Law, University of Pittsburgh, Pittsburgh, PA, United States; ^2^Charles Widger Endowed University Professor, Villanova University Charles Widger School of Law, Villanova, PA, United States; ^3^University of Illinois School of Information Sciences, Champaign, IL, United States; ^4^Alfred E. Engelberg Professor of Law, School of Law, New York University, New York, NY, United States

**Keywords:** abundance, scarcity, knowledge commons, social dilemmas, institutional analysis, information, data

## Abstract

The economics of abundance, along with the sociology of abundance, the law of abundance, and so forth, should be re-framed, linked, and situated in a common context for empirical rather than conceptual research. Abundance may seem to be a new, big thing, between anxiety over information overload, Big Data, and related technological disruptions. But scholars know that abundance is an ancient phenomenon, which only seemed to disappear as twentieth century social science focused on scarcity instead. Restoring the study of abundance, and figuring out how to solve the problems that abundance might create, means shedding disciplinary blinders and going back to basics. How does abundance, in various forms, create or alleviate social problems? We explain and illustrate how the Governing Knowledge Commons (GKC) framework provides a useful research tool to generate and test hypotheses about abundance in various economic, social, cultural, and legal settings.

## Introduction

Consider again the lobster, to borrow the title of Wallace's (2004) well-known essay. Though lobster meat was once so abundant that it was deemed suitable only for the poor, in the twentieth century trapping lobsters started to replace plucking them straight from shallow water. Only then did lobsters become symbols of status, taste, and value (Mishan, 2021). And governance. Acheson's (1988) “The Lobster Gangs of Maine” remains a seminal study of community management of depletable lobster fisheries, an anchor and pole star for research in the tradition of Elinor Ostrom. The problems of “too much of a bad thing” were turned into the opportunities for productive resource management that Ostrom associates with communities and collectives.

We argue that the spirit of Acheson and Ostrom should be directed generally to abundance problems in twenty-first century economics, society, and culture and specifically to abundance problems surrounding the most critical social challenges of the present, those relating to knowledge, information, and data. Theory should be modest. Careful, contextual, systematic empirics should inform it. We illustrate that argument with examples drawn from applications of the Governing Knowledge Commons research framework.

Part 2 of the article explains what we mean by abundance and points out the limitations of the standard abundance vs. scarcity framing in twentieth-century writing. Part 3 lays out our different framing, including the importance of context and empirical understanding. Part 3 links that framing to a review of social dilemmas that are particularly apt to appear in resource contexts labeled “abundance.” We show how the empirical research that we envision can be undertaken using the Governing Knowledge Commons (GKC) research framework. Part 4 reviews studies of knowledge commons governance in the context of information abundance as use cases for the GKC framework. Part 5 concludes with implications and recommendations for future research.

## Information Abundance and Why Governance Matters

The most compelling and easiest to grasp sources of resource abundance today come from fields we associate with knowledge, information, and data. A quick search for the phrase “information overload” turns up numerous popular and scholarly treatments of various Internet systems and platforms, social media, and the many challenges of mis-information and dis-information. The phrase “Big Data” is overused as shorthand for the seemingly overwhelming volumes of digital data collected by companies, governments, and nonprofit organizations. The overuse is telling. Data are ubiquitous and important. Practical and political questions concerning data abundance are abundant. Consider emerging rhetoric surrounding “the metaverse.” If we can create one metaverse, why not a second, a third, and so on?

Meanwhile, the most urgent environmental question facing the planet in the twenty first century is partly a matter of physics and chemistry, as carbon dioxide emissions dilute a seemingly-abundant resource—the Earth's atmosphere. Look more closely at the problems we associate with climate change, and one sees not only the interaction of abundant CO2 and a depletable pool of unpolluted air but also a coordination problem involving nearly innumerable sources of key political, scientific, economic, and cultural capital.

What do these anecdotal examples have in common? The answer is, we argue, a widely-shared intuition that solutions to these problems lie not with standard responses grounded in the idea of scarcity. Can we identify scarce resources or create resource scarcity in order to eliminate or mitigate the harms caused by information overload? By Big Data? By the knowledge- and expertise-politics of climate change? At best, that strategy is incomplete.

To explain, this section lays out what we refer to as the abundance hypothesis, both in a casual or colloquial form and in what we understand to be its more technical contemporary sense. Regardless of how the hypothesis is framed, we argue that it leads to unproductive if not altogether wrong responses and results. We lay those out, too, briefly, so that our (different) response is put in proper context.

### The Abundance Hypothesis and the Standard Responses

“Abundance” and “scarcity” have both have multiple meanings. We review them below, because what we describe as the abundance hypothesis depends on some conceptual categories. But that's where the utility ends. We argue that conceptual categorizing is misleading.

#### The Hypothesis

In a nutshell, the abundance hypothesis is: You can't have too much of a good thing. Concretely and more carefully, the hypothesis is that if (or, sometimes, since) abundance is the default condition of twenty-first century resource systems, the regulatory infrastructures that defined the scarcity economy are unneeded and should be updated and replaced where appropriate.

Let's break this down in different respects. The hypothesis pushes us toward some definitional questions, or what we call the ontology of abundance.

Abundance relates to a resource, or to resources.

What's a resource? Resources are potentially usable or useful inputs to social and ecological systems. They may be objects, material or immaterial, but they don't have to be. The fact that they're potentially useful doesn't mean that they ought to be used. Sometimes, their best use involves leaving them mostly alone. Sometimes, calling objects “resources” tends to treat them as “things” or as subjects of human control in ways that conflicts with ideological or philosophical intuitions. We leave those debates for another time and place. The word “resources” here is just a starting point, and it's a broad one. Resources may be biophysical, because they're produced by mostly natural processes. They may be cultural, because they're produced by mostly human-directed processes. They may be material (books) or immaterial (creative works), bounded (trees) or unbounded (air), living (animals) or not (rocks). Resources are subject to a host of definitional and characterization issues, because we often have difficulty figuring out how to define and describe what a resource or a thing “is” or “should be” (Madison, 2005; Fennell, 2019). Often, more important than the resource itself is the fact that resource units, such as trees, or books, are parts of resource systems, such as forests, or libraries.

What's abundance? Economists speak in one language. Information technologists speak in a second. Sociologists and students of culture speak in a third. Legal scholars speak in a fourth. Casual or common usage is a fifth. We're pluralists; each of these is relevant, even if they're relevant in somewhat different ways.

Economists distinguish between private goods (rival, depletable, excludable) and public goods (none of those). They also speak of club goods (public goods to the members of a community, private goods to others). The conceptual intuition here, which often animates public policy debates about information law, such as copyright and patent, is that the intangible innovative or creative content in a machine or a book is a public good and the physical object is a private good. Patent and copyright exist to supply artificial scarcity to what would otherwise by harmfully abundant knowledge. (Paradoxically, perhaps, the artificial scarcity is intended to motivate the production of more knowledge.) In a related vein, economists situate abundance in the context of supply and demand. Twentieth century welfare economics exists largely to answer the question: because resources are scarce, how should they be allocated? If demand for a good can be fully satisfied with resources to spare, which is to say that no need is unmet, then the resource is abundant.

Information technologists often build on distinctions between mostly material things (presumed to be scarce) and mostly immaterial things (often presumed to be abundant). The supply of digital objects, such as data, software code and apps, is presumed to be limitless, and therefore abundant, because it may appear that those objects can be produced and shared nearly effortlessly, sometimes even unintentionally, and by nearly anyone, as long as one can scale up the necessary power, processing, and storage capacity. In practice both the supply and the scaling up do have limits, in their origins and effects in the material world, in social organization, and in individual experiences with information and data.

Sociologists and students of culture look at abundance as a matter of human practice and look for social patterns. Resource abundance or scarcity is less a matter of materiality and more a matter of wealth, status, and perception. Do people have enough? Do they have too much? Do they have an excess, or a surplus? Are the resources made by people or by nature? Each of those questions may underlie judgments labeled “abundance” or “scarcity” (Cohen, 2017; Boczkowski, 2021).

Lawyers and legal scholars have no standard syntax for abundance and scarcity, despite their field's typical concern with definitional precision. Instead, practitioners and researchers in different legal domains borrow as needed from their social science cousins, usually recognizing that the character of a resource in most respects is a function of social processes of construction and interpretation. Rose (1990) argues persuasively that property resources are constructed and managed *via* stories. Rakoff (2002) examines one of the most abundant natural resources—time—and finds it subjected by law to numerous contortions to suit human purposes.

Casual or common usage matters, too, because in day-to-day usage, “abundant” typically means “a lot”—a lot of people, a lot of stuff, a lot of time, and so forth. That might mean “a lot” relative to what came before; it might mean “a lot” relative to future expectations; it might be “a lot” relative to whether the counting might ever stop. This sort of thing appears especially in contemporary popular accounts of internet infrastructures, and until recently it carried the aroma of awesomeness, as in the writings of Jeremy Rifkin (“The Zero Marginal Cost Society”) and Clay Shirky (“Here Comes Everybody” and “Cognitive Surplus”) (Shirky, 2008, 2010; Rifkin, 2014).

#### The Standard Responses

In Shirky's writing, the modern ontology of abundance turns out to lead almost always and almost directly to a party. Reward! Opportunity! Growth! Health! There's no such thing as too much of a good thing, largely because with so much of a good thing, we don't need the government to step in to supply it, or to regulate it. If distributional problems are evident, we can manage them by tweaking the system. Over-optimism remains characteristic of many contemporary critiques of scarcity thinking. In the late 1990s, early Internet idealists like Stewart Brand and John Perry Barlow celebrated the release of material shackles from newly-digitized information. Only slightly less manic reactions came from libertarian-minded legal scholars, who claimed not only that traditional terrestrial governments lacked the power to control and regulate abundant digital information, but also that this powerlessness was a good thing, too (Johnson and Post, 1995). Benkler (2006) early work on commons-based peer production pronounced the triumph of abundant distributed cognition over centralized systems for producing information goods. Today's blockchain enthusiasts subscribe to an equivalent philosophy: there are no functional limits to the length of append-only digital ledgers. More blockchain is better.

What links the responses? The celebratory conclusion that abundance means that the justifications for law and policy dilemmas in a scarcity-based economy and society are no longer operative. A scarcity-based economy and society needs lots of coordination by the state and lots of regulation, to make sure that resources are produced in the first place and to make sure that those resources are distributed appropriately in the second place—perhaps efficiently, perhaps equitably, perhaps in some other way. The scarcity economy is defined, in simplistic terms, by one version or another of the coordination problem that Hardin called “the tragedy of the commons” and that was sketched by many others, including Olson (1965). A depletable resource will be overconsumed if access to the resource is not defined and managed by some central authority, such as the state. Because of the overconsumption, the resource may be underproduced. Who would produce a thing when anyone can come in and walk away with as much of it as they wish?

Abundance eliminates the tragedy of the commons. In Rose's (1986) felicitous phrase, with an abundant resource, we have a comedy of the commons. When resources are abundant—either because we make so many of them (such as information) or we find so much of them (such as sunlight)—over-consumption doesn't dampen production. We don't need governments to prompt production. No matter what we do, there's plenty. And we don't need the state to steer allocation. Everyone gets at least as much as they need, and perhaps more. Maybe we need the state to supply guardrails to ensure health, safety, and environmental stewardship. Maybe the state is needed to supply conflict resolution services where disputes over resource access or allocation arise, and to ensure that private markets for resource re-distribution function effectively. But abundance leads, plausibly, to a state that is considerably smaller than the state pre-supposed by resource scarcity. Less law. Less policy.

This overview is obviously simplified and stylized. Importantly, it's a sketch of a series of interrelated concepts, not a description of the world as it is. Yet it captures the celebratory tone that often accompanies the abundance hypothesis. Scarcity is over; we should worry less.

### Why the Abundance Hypothesis and the Standard Responses Are Wrong

The celebration shouldn't begin just yet. No matter the language, abundance may be real (or may not, as we'll see), but its causes, uses, and effects are, as so often turns out in the real world, complicated. The specifics of the abundance hypothesis, as a species of abundance theorizing generally, go back centuries. There really is nothing new under the sun. (Of course, sunlight is one of the most abundant resources that we know.) When we look more carefully at what abundance means and what it represents, we uncover a mixed portrait that blends both abundance and scarcity, celebration and concern.

Examples abound. Begin with history, in which abundance was sometimes viewed as a reward and sometimes as a concern, even as part of the usual order of things. In the Old Testament, the blessings of abundance were promised to those who built the Second Temple of Jerusalem. The foundations of classical economic theory were laid not by those who worried about how to manage scarcity but instead by those who worried about how to manage collective wealth. Adam Smith's treatise of 1776 was titled “The Wealth of Nations” and was concerned with, among other things “the different Progress of Opulence.” The blessings of abundance were at last realized by early industrialists, or so they thought. Marx theorized the origins of capital as surplus value extracted from labor. The ideologies of the consumer in early market capitalism drew Veblen's critique in “The Theory of the Leisure Class” in 1899.

As recently as the 1950s, some economists looked at global economic systems partly in terms of how to use productively the West's (and particularly the United States') massive accumulations of surplus wealth (McGoey, 2018).

Over the last decade, Piketty (2014), among other scholars, introduced the possibilities that abundance and its possible cousin, inequality, should be restored to places of prominence in the canons of economics. But Piketty is hardly a celebrant of abundant wealth in the modern era. He highlights its downsides. Similar calls to restore significant attention to the problems caused by abundance come from sociologists (Ritzer and Jurgenson, 2010; Abbott, 2014), anthropologists (McCracken, 1998), geographers (Hoeschele, 2010), and legal scholars (Johns, 2013; Desai, 2014). In different respects, each of them recognizes that abundance might be celebrated but need not be, and that recognizing abundance in a particular social, cultural, or economic context requires thoughtful recalibrating of what it means to recognize some resources as plentiful and some resources as scarce.

This quick account of the intellectual history of abundance is all too brief but drives home a single point: the abundance hypothesis goes wrong in assuming that the tragic commons and similar collective action dilemmas exhaust the inventory of social problems associated with managing a resource, whether it's scarce or abundant. That's true whether we're considering law or public policy or other institutional forms. The disappearance of tragic commons dilemmas does not solve all social problems associated with producing and managing resources. Current scholars imagine that a new social science of abundance is needed to take advantage of abundance, because some combination of technology, societal forces, and/or law have changed the stakes of scarcity. Prompted by questions surrounding intellectual property law, for example, Lemley (2015, p. 515) concludes, “[u]nderstanding what a post-scarcity economy will look like is the great task of economics for the next century”. That statement stops short of asking: what are the social problems associated with abundance—even after we acknowledge harmful externalities and spillovers, such as health, safety, and environmental concerns?

When we focus directly on knowledge and information as key abundant resources, history teaches that we should be careful what we wish for. Even abundance has its dark sides. Sometimes abundance offers under-recognized problems and possibilities. Two particular gaps in the abundance hypothesis should be called out as foundational reasons to doubt its general wisdom: its focus on materiality and thing-ness and its focus on markets and states. The next Section explains how those opens the door to exploring additional social dilemmas of abundance.

#### The Mistaken Focus on Materiality and Thing-Ness

The abundance hypothesis looks at the world in terms of stuff, and in particular in terms of objects. That focus can be misleading, particularly when attention shifts back and forth uncritically between material stuff and immaterial stuff. The language of resources sometimes contributes to confusion here. The word “resources” itself can be taken intuitively to refer to depletable stuff (water) or rival things (physical objects) rather than to inputs into social systems. To put that point somewhat more technically, a resource is something for which there is social demand (Frischmann, 2012).

So, when the abundance hypothesis examines the modern world, and in particular looks at the modern worlds of data, information, and knowledge, it assumes that resources are material or immaterial. Material resources are physically scarce (depletable or rivalrous or both), while immaterial resources (naturally non-depletable, non-rival) must be shunted into legal forms of scarcity (patents, copyrights, and so on) in order to ensure their production. As information circulates today more in digital (presumptively immaterial) forms and less in material forms, the technological drivers of scarcity fall away, leaving only questions about whether artificial scarcity can still be justified. Books and the like (and inputs into them, such as paper and ink) are naturally material and therefore scarce. Informational “things,” such as inventions and creative works, are naturally intangible and only unnaturally object-like.

That story simplifies the matter far too quickly and easily. It has a pseudo-ontological basis. In law and economics, Brett Frischmann has described how the propensity toward using scarcity as a one-size fits all solution to resource management problems is just as unfounded as a naïve celebration of abundance (Frischmann, 2007b). That point is illustrated by the fluidity of lines between the material and immaterial. Those lines are mutable to a significant degree. They are often affected by both nature and human activity. And their impacts are nuanced, based on social and cultural context. Science and Technology Studies (STS) scholars have documented the multiple ways in which lines between conceptual intangibility and tangible manifestation and object-ness have been manipulated and constructed by social processes (Bijker, 1995; Latour, 2007). We need not embrace any particular strand of STS theory or research to make that point. Both histories of objects (Petroski, 2006) and histories of intellectual property (Op den Kamp and Hunter, 2019) show the dense interweaving of the material, scarce “thing” and its sources and the immaterial, abundant “idea of the thing.” That interweaving evolves both over the long time scales of cultural evolution and in the moment of specific conflicts over ownership, use, and meaning. As the historian of science Galison (2018) has shown, even in research science, ontological approaches are beginnings rather than endings, because you can always steer an “abundance” problem into a constructed “scarcity” problem.

Does the tangible/intangible mutability problem operate in different and perhaps simpler ways for information than for systems grounded explicitly in material resources? Some brief history shows that it doesn't.

Start with copyright. Conventional modern wisdom holds that copyrighted works need to be fenced off (that is, made scarce) to motivate production and distribution of creative things (possibly knowledge things, but the difference doesn't matter here). Without fencing, prices reflect marginal (competitive) cost; authors and publishers are unable to recover the fixed costs of production and therefore won't invest and produce new works. We have too few books, in other words, so we produce artificial scarcity to get more of them. Copyright declares that we want “more” of the creative and intellectual content that the books “contain.” Copyright purports to solve an anticipated scarcity of immaterial content by creating an artificial scarcity of material books. But copyright policy is mostly indifferent to the particular books we get. It fails to recognize that the production of content has other facets involving other sorts of social dilemma. Society may not want merely “more” and more books of whatever sort. Slightly different versions of this story apply now to things as diverse as feature films, popular music, poetry, photography, videogames, and computer programs.

It turns out that the plot of this story is precisely the opposite of the plot of the story that justified knowledge regulation centuries ago, in Enlightenment Europe. But the scarcity and abundance characters are the same. The historian Chad Wellmon explains: Back then, the problem wasn't too few books. The problem was that the world had too many books, a product of new printing technology. A modern observer might wonder what the problem was. But deeper underlying epistemological conditions were different. Knowledge was believed to be universal, the duty of an enlightened person was to know, and to know meant to know everything. Knowledge only counted as such if it was knowable by all. With the proliferation of books, the amount of knowledge on offer expanded, and it expanded too quickly for learned people to conclude that they could know everything (Pasanek and Wellmon, 2015; Wellmon, 2016).

Wellmon (2016) observes that the university emerged as a regulatory solution to this knowledge production problem. At that macro institutional level, universities organized knowledge into disciplines and faculties, with material and immaterial constraints on participation, precisely to address the Enlightenment version of information overload—i.e., abundance. If it wasn't possible to know everything, it became possible to know everything in one's field or discipline. Both internally in the university environment and externally as members of that system engaged with people outside of it, university organization changed what it meant to be “learned.” In that institutional context, epistemology and culture eventually worked out the content (potentially abundant) vs. container (usually scarce) distinction that evolved into the foundation of modern copyright. The content was the knowledge of interest to scholars; the container was merely a commercial object. Wellmon's explication of the history of universities is consistent with recent work exploring the university as a knowledge governance institution (Madison et al., 2009).

At a micro level, related historical trajectories show how research practices and techniques of information organization seemingly solved overload problems in the lab and in the library. Linnaeus's classification system for living things owed its success in part to his heavy reliance on index cards (Krapp, 2019). The Dewey Decimal system, indexing practices, and other knowledge classification systems changed how library books were shelved and used (Blair, 2010; Burke, 2014; Duncan, 2022). Complex relationships between information organization and classification, on the one hand, and social practice, on the other hand, is a robust and thriving field of research and practice (Bowker and Star, 1999; Glushko, 2013).

None of that is to suggest that any of these institutions or practices, material or immaterial, ever have been comprehensive solutions or problem-free. Wellmon's research is part of a revival of research and practical interest in the futures of universities. Scholars and researchers still only have twenty-four days and limited attention with which to consume information, despite its abundance. Twenty-first century information intermediaries such as Google, which originated in the instinct to help Internet users navigate information overload on the World Wide Web, have generated some of the most challenging information production and distribution challenges of the present day (Cohen, 2019). They are, in many ways, sources of abundance problems that cannot be addressed in simple abundance vs. scarcity terms.

#### The Mistaken Focus on Markets and States

The focus of the abundance hypothesis on material things feeds into a related but higher order focus on how law, economics, and related regulatory thinking should advance. In a market economy, things are expected to be produced and circulated in markets, *via* voluntary, bilateral transactions. Sometimes markets under-perform, either because expected production and distribution doesn't take place or because production and distribution cause harm. The state is expected to step in and do one of two things: fix the market so that it works “better” or figure out how to ameliorate the harm, or both. So long as things are scarce, either naturally or artificially, that general equilibrium seems to hold, at least as a conceptual matter. The abundance hypothesis tends to celebrate because this focus on markets and states makes it appear that in a world of resource abundance, the role of the state can simply be more limited. That's too narrow a view.

Here we draw on the research of Elinor Ostrom, who was awarded the Nobel Prize in Economic Sciences in 2009 for her work on resource governance and in particular for her demonstrations that “the market” and “the state” are not the only two governance modes for successfully addressing resource management challenges in a given community setting. Other governance institutions can and do exist and perform effectively. She described these as community-based. The research world justifiably sees her career as responding definitively and empirically to the conceptual challenge raised by Hardin and the “tragedy of the commons” metaphor and simplifies her contribution in the phrase “commons.” Looking at Ostrom a bit more carefully draws out some important details. Ostrom sketched a type of resource that she added to economists' standard inventory: common-pool resources (depletable but non-excludable, and therefore shared). She showed that the tragic commons was not an inevitable result of resource use by multiple actors. She demonstrated empirically that these common-pool resources could be produced and maintained sustainably by local communities. Those communities governed themselves largely by principles that she documented in her foundational book, “Governing the Commons” (Ostrom, 1990) and elaborated on in “Understanding Institutional Diversity” (Ostrom, 2005). Critically, she insisted that analysis and answers needed to proceed carefully and contextually. There was no one-size-fits-all solution.

Ostrom (2010) titled her Nobel Prize lecture “Beyond Markets and States” precisely because in many respects her signature contribution to institutional analysis was not a specific focus on commons institutions and resource management as such, but instead consisted of opening and documenting an important perspective on institutional governance in complex settings. If we have too few resources and want more, or have too many resources and want fewer, or otherwise want to deal with expected and unexpected by-products of resource systems, reinforcing markets and empowering the state are not the only options. And, in the recursive way in which Ostrom's work is always best understood, expanding the range of institutional solutions also expands the ways in which social dilemmas are identified and framed. Closely related to her work on institutional governance was her interest in polycentricity, accepting the inevitability and sometimes the value of governance systems that are multi-modal with respect to sources and spheres of power.

### Governing Knowledge Commons: A Broader Perspective

Does Ostrom's view of complex polycentric order and the expanded universe of institutional governance operate differently when it comes to information and knowledge resources? After all, her arguments about commons governance were drawn largely from studies of natural resources, such as forests, water resources, and fisheries, and her addition to the economists' toolkit of goods was “common pool resources,” which she defined as depletable things. As Acheson pointed out, a lobster fishery can be depleted over time, through overfishing. What does Ostrom have to do with abundance, where depletability, and the tragedy of the commons, are not dominant concerns?

We argue that Ostrom's intuitions about empiricism, context, and an expanded, pluralist view of the institutional landscape should be brought to bear on the challenges and opportunities associated with abundant resources (Frischmann, 2013). We focus particularly on abundant information and knowledge resources. Many of the specifics of Ostrom's program, including her taxonomy of goods, her research frameworks, and her enthusiasm for polycentricity, are less useful in the information and knowledge setting. We have built on the intuitions and constructed our own intellectual framework and research approach, the Governing Knowledge Commons (GKC) framework, which we argue has been and should be applied broadly to understand and diagnose the character of abundance in social context. Part 3 describes the GKC framework and its purposes. Part 4 illustrates how the framework has been used so far to capture significant attributes of abundance in particular resource settings.

## Abundance, Context, and Governance: Using the GKC Framework

The Governing Knowledge Commons research framework builds on a series of intuitions, beginning with the premise that information, knowledge, and data resources are different because of their presumed intangibility. Are they in fact abundant, within any of the meanings of “abundance” mentioned earlier? If they are (or even if they are not), how do we identify and catalog the social problems and solutions that “abundant” information offers? The goal is to build a systematic investigation rather that to rely solely on storytelling and simple stereotypes (McAdams, 2009). If abundance celebrations are premature, then what takes their place? Whether scarce or abundant, information and knowledge don't govern themselves. What governance do we see, what do we not see, and what explains both what's there and what's not?

### Hypothesizing the Dilemmas of Abundance

We begin with some speculations, to prime the pump for the detailed follow-up research that we believe is needed. The question that we begin with—“what social dilemmas are implicated by resource abundance, or by shifts from resource scarcity to resource abundance?”—is a core part of the GKC research strategy, as we describe in the next Section. GKC-focused research investigates cases of information governance as responses to social dilemmas. We call out social dilemmas separately, as hypotheses to test, in order to make the case that the GKC framework provides a starting point for synthesizing this research systematically. Otherwise, exploring “abundance” falls back either into intellectual and disciplinary silos or into case-specific problems with non-transferable solutions.

By “social dilemma” we mean, mostly, a context-specific conflict between individual welfare and social welfare. A social dilemma is often described as a conflict between rational choosing at the individual level and the product of rational choice at the collective level. The metaphorical tragedy of the commons fits that model, as one prototypical collective action and coordination dilemma. Our use of the phrase “social dilemma” is not constrained to rational choice expectations or to the premise that we are exploring only choice-directed activity. Individual and collective action in the real world is obviously subject to behavioral and cognitive constraints. Welfare at many levels is subject to various historical contingencies. Modeling resource governance as a successful or flawed product of rational behavior is analytically simple, comparatively speaking, but descriptively incomplete. GKC-based research aims at descriptive completeness. Specifying social dilemmas—plural—is a way of specifying what contexts matter in understanding problems and solutions.

We also don't limit GKC research and the character of relevant social dilemmas to those specified by economic frameworks for resource design, distribution, or allocation. Or those predicted by legal understandings, sociological theories, historical narratives, or any other single disciplinary perspective. The GKC framework is intended to be open to adoption and use by researchers from many different traditions, using any necessary translations into appropriate conceptual syntax. The language of supply and demand should be expected to bump into the language of participatory democracy, the language of ideology and social meaning, the language of power and influence, and so on. Next steps could then include conceptual modeling, computational analysis, experimentation, and qualitative evaluation.

Our inventory of abundance-related social dilemmas consists of the following. We believe that it's a good starting inventory, but we don't contend that it's the last word. We offer these as a series of hypotheses, with special attention to information and knowledge abundance. Obviously not each hypothesis will be relevant in each context, and where relevant, some hypotheses will be interconnected. They vary considerably in terms of the level of governance generality that each one addresses. Some focus on more abstract system-level or group- or community-level concerns. Some focus on more concrete considerations related to the specific use of a given resource.

(1) Information abundance sharpens and highlights conflicts in classic social theory that emphasize either the role of structure and system, on the one hand, or individual agency, on the other hand, in producing system outcomes. Amid abundant information, how do individuals distinguish themselves, positively and negatively?
(2) The sources and impacts of information abundance are interwoven with the sources and impacts of resource scarcity, rather than independent of them. How are the benefits and harms of information abundance enhanced or ameliorated by the fact that information is usually anchored in physical systems and things?
(3) Sources and impacts of information abundance are based significantly on spillovers from and to other resource systems, challenging assumptions about context and consistency in interpretation. If information is everywhere, at least conceivably, then presumably it easily affects people and systems for whom it wasn't designed or intended.
(4) Information abundance may produce or reflect creative production, but it also may produce or contribute to cultural or social stagnation, in that individuals may have little reason to choose among different resources or resource sets. This hypothesis includes investigation of information congestion and information waste. If people have everything that they seem to want or need, how are they motivated to improve themselves or their communities?
(5) Information abundance may create or contribute to cultural or social dis-equilibrium, in that individuals may be cognitively or emotionally incapable of choosing among different resources or resource sets. There may be too much information to pay attention to effectively and no stable value-based frameworks to use in setting priorities. In a world of information overload, how do we identify what matters and what is true?
(6) Information abundance may obscure possible social tradeoffs among information quality, such as producing and distributing better information; information quantity, such as producing and distributing more information; information balance, such as healthy diversity; and information equity, such as ensuring fair access to and capability to make uses of information. Sometimes those tradeoffs arise from ordinary or customary information practices; sometimes they arise from purposeful, even strategic information and misinformation practices. Does information abundance make it easier or harder to implement and reconcile different interpretations of the promise of cultural progress?
(7) Information abundance exacerbates the complexities of participatory governance in collective or community settings, making both effective participation in relevant communities, but also exit, more difficult. How does information overload affect not only understanding but also social engagement?
(8) Information abundance increases the importance of reputational stakes as governance mechanisms in a given context. Are information “goods” necessarily so-called “Veblen” goods, at least in part, in the sense that they are valued for their use in signifying social status?
(9) Information abundance (expands) (diminishes) conceptual spaces for the effective operation of privacy and free expression practices. In a world of abundant information, how does a community identify, implement, and maintain appropriate systems to advance interests in individual privacy and personal autonomy?
(10) Information abundance creates demands for intermediary systems to organize information and knowledge across space, time, and community, so that information can be rendered accessible and usable and so that different bodies of information can be articulated relative to each other. How does knowledge abundance create demands for more knowledge?
(11) Information abundance creates demands for intermediary systems to provide education and other capabilities to enable individuals to access and use that information. How does knowledge abundance create demands for how to know?
(12) Information abundance (enhances) (diminishes) possibilities that information ecosystems will evolve in complex ways and will produce unplanned, emergent order. When and how does information abundance produce information or other products that we didn't plan for or expect, for good and for ill?

### The GKC Framework as an Approach to Empirical Analysis

The origins of the GKC research framework lie in several intersecting trajectories of research and analysis on institutions of knowledge governance. One is disappointment with the anecdotal, a-systematic character of research on community-based innovation and creativity, a body of research dubbed “IP without IP,” or intellectual production without intellectual property (Dreyfuss, 2010). Two is interest in ecological and systems perspectives on knowledge, culture, and intellectual resources, also anchored initially in analysis of intellectual property law (Madison, 2005; Frischmann, 2007a). Three is Ostrom's work itself; toward the end of her career, Ostrom, with Charlotte Hess, turned her attention to the possibility that knowledge resources might be a good object of Ostromian study (Hess and Ostrom, 2005; Hess, 2012). Four is information science and management studies, which in different respects have extended their traditional interests in the organization of knowledge to embrace community-centered perspectives, including Ostrom (von Hippel, 2005; Borgman, 2015).

That intellectual pluralism necessitated the development of a research strategy that is suitably flexible yet also capable of yielding systematic results over time, as Ostrom's has been. The GKC framework is modeled in part on Ostrom's Institutional Analysis and Development (IAD) research framework. Where the IAD framework is directed principally to exploring governance mechanics relative to biophysical materials organized as Ostromian common pool resources, the GKC framework begins by querying rather than assuming the characteristics of the resources at stake in some knowledge or information governance system. Because those resources are almost always blends of immaterial and material conditions, affected in different ways by relevant legal systems (such as patent and copyright), the GKC framework calls for careful delineation of the interplay between resource attributes and social dilemmas. Multiple sorts of resources may be circulating simultaneously in a given social system, and that system may be characterized by multiple social dilemmas. Institutional pluralism, including community governance alongside market-based systems and state-dictated systems, may be particularly important in systems that operate at multiple levels simultaneously.

The shared character of at least some of those resources, usually presumed because at least some of them are “open” in one respect or another, is often a useful starting point for further description. That shared character is also usually the basis for referring to the governance system as “a commons.” More precisely, GKC analysis uses the phrase “knowledge commons” to refer to governance rather than to the resource. Governance refers to groups or communities of people who share access to and/or use of the resource and who manage their behavior *via* an established set of formal and informal rules and norms. Commons are distinguished from non-commons by the institutionalization of sharing of resources among community members (Madison et al., 2010).

That definition points the way to the further steps in a GKC-based case study. The community or collective setting (or settings) in which information and knowledge is produced, stored, and/or circulated should be defined and described. How are members or other participants identified, permitted to participate in governance (or excluded), and what sorts of roles or hierarchies—informal or formal—describe their interactions? Recent elaborations of the GKC framework highlight the contributions of theories of democratic participation to GKC study, including, per Hirschman, the relevance of exit, voice, and loyalty options to community members (Sanfilippo M. et al., 2021). These community settings or contexts may be defined as “action arenas” per Ostrom's vocabulary, with more or less porous or dynamic borders and boundaries and context defined culturally, economically, legally, and/or physically.

Context is, in this sense, more than the environment in which a social dilemma occurs. Context is the combination of social, cultural, psychological, historical, political, economic, physical, and technical factors around the challenge of interest. With respect to social dilemmas around information and technology, context is the entirety of the specific, complex, and dynamic sociotechnical systems in which people engage with those technologies or that information (Kling et al., 2005). Context matters because we don't engage with technology or information within a vacuum. Context shapes our expectations and interpretations of information, as well as their flows among people and systems (Nissenbaum, 2010).

Within those action arenas, formal and informal rules, customs, norms, and expectations define not only the resources themselves but also how they are created, accessed, replenished, and combined with other resources. These “rules in use,” again to borrow from Ostrom's language, may also include mechanisms for policing appropriate behavior and for resolving disputes.

Each of these topics can be clustered in a “bucket” of questions for research, so that there is no set sequence or priority for any one of them either as a matter of research strategy or as a matter of analysis. The interdependency of the results matters as much as the data collected in each case. Both conceptually and in practice, beliefs and behaviors that may be categorized in one way end up both affecting and being affected by beliefs and behaviors put in other categories. A given case study may opt to focus on one or more of these buckets to the exclusion of others.

**Figure 1:**
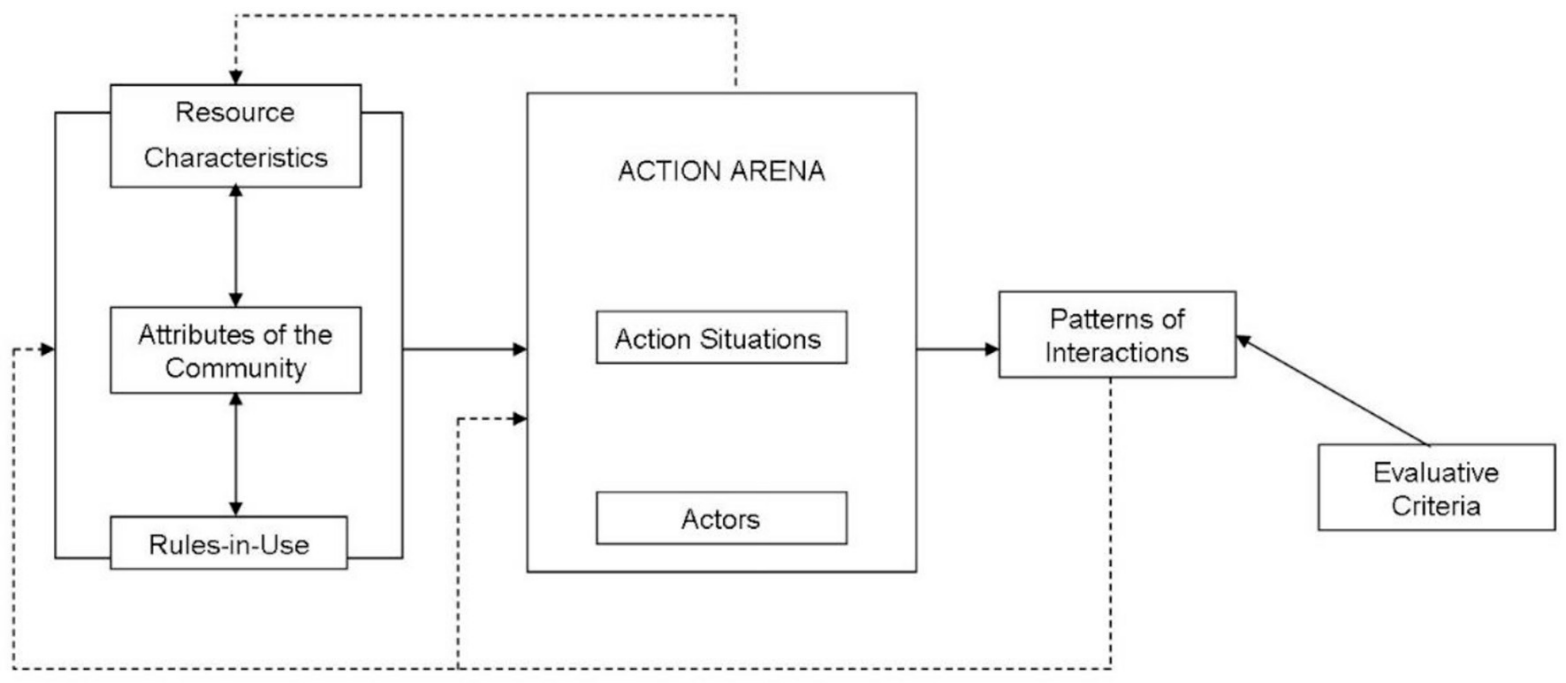
The Governing Knowledge Commons (GKC) Research Framework. Source: Madison et al., 2010.

A schematic of the GKC framework appears as Figure 1.

That schematic highlights the likelihood that the outcomes of GKC research consists of identifying patterns of social interactions both as a key payoff of an information governance system and as a key input into the continuing function of that system. That's a key difference between GKC analysis and IAD analysis, which focuses on the sustainable production or maintenance of biophysical resources themselves. And it exposes the part of the GKC field that is the least developed so far: how to subject the results of this descriptive analysis to meaningful normative analysis? In information and knowledge domains, as the earlier list of information abundance hypotheses suggests, competing and overlapping normative criteria are abundant.

It seems plausible that criteria for assessing resource allocation in a conceptual world dominated by scarcity—various modes of economic efficiency; utility; and distributive justice—are at best only partly relevant in contexts characterized wholly or partly by abundance. One of us (Frischmann) has suggested that a human capabilities approach may offer a promising alternative. That strategy is also wanting in certain key respects. Once capabilities to participate are fully described and assessed, does it matter how rules for participation in governance distribute those capabilities? Does it matter whether the results of an information system, particularly a system characterized by information abundance, are in some meaningful respects accurate or true?

We can't resolve those questions here. We note that the questions can and should be raised in GKC-directed case studies. We anticipate that the GKC framework has a long way to go in framing future case studies and additional empirical work.

Most important to this article, we note that the GKC has a track record, which documents its steady progress toward not simply adoption and use but toward utility as a research device. What is now known as the GKC framework was launched in 2010 in an article titled “Constructing Commons in the Cultural Environment” (Madison et al., 2010) and has since been elaborated *via* three published collections of case studies overseen by the authors of that work and various other research, some produced under the umbrella of what is known as the Workshop on Governing Knowledge Commons (https://knowledge-commons.net) (Frischmann et al., 2014, 2017; Sanfilippo M. R. et al., 2021) and some produced by researchers working independently (Dekker and Kuchar, 2021).

## Illustrations: The GKC Framework and Governing Abundance

The GKC framework is best understood in case-specific context, just like the governance that it tries to describe. Some cases involve small communities. Some involve large, distributed collectives. Some are grounded in volunteerism, some in market capitalism. Some involve infrastructural resources. Some focus on finished products or consumer-facing services. There is no single standard or paradigmatic case of knowledge commons. That's precisely its strength. If information and knowledge are everywhere in the economy and society, the framework has to be flexible enough to capture that diversity. It is. Here, we've included brief descriptions of completed case studies in the GKC portfolio that illustrate specifically how the framework illuminates problems and solutions in cases of information or knowledge abundance. For additional examples, see “Information Abundance and Knowledge Commons” (Madison, 2016).

### Universities

The first case study published by the authors of the GKC framework focuses on the university itself, as a knowledge-producing and knowledge-storing institution with an extensive history, lots of institutional diversity within the overall conception of “the university,” and enormous current critical intellectual, economic, political, and cultural stress. As noted above, the Enlightenment antecedents of modern research universities emerged precisely to address then-current problems of knowledge overload, a species of knowledge abundance. The sociologist Andrew Abbott brings those concerns with information overload up to date as matters of personal identity and social structure (Abbott, 2014). Kitchin (2014) makes a similar contemporary argument as a matter of epistemology. “The University as Constructed Cultural Commons” documents the histories of universities as governance institutions for knowledge sharing, noting the complex interplay of knowledge resources, the purposes of universities, and the various material forms that define universities today (Madison et al., 2009).

### Citizen Science

“Commons at the Intersection of Peer Production, Citizen Science, and Big Data” explores governance of a citizen science project called Galaxy Zoo. Galaxy Zoo was launched in 2007 initially to aid some University of Oxford researchers in classifying massive volumes of astronomical data. An abundance of galaxies, to be specific (Madison, 2014). The project directors began with modest ambitions, understanding that hand-based classification by experts would never be sufficient to complete their research task and hoping that amateurs, with modest guidance, could do it as well or better *via* the Internet. The leaders were nearly overwhelmed by the rapid positive uptake of the system they built. Their galaxy classification research project evolved into the formal “Galaxy Zoo,” with spinoff citizen science projects, formal but inclusive governance practices, and some interesting and useful knowledge spillovers as some volunteer “Zooites” converted their early informal engagement into longer term research programs of their own.

### Biobanks

A different domain of scientific research, biobanks, offers an interesting contrast in managing enormous volumes of knowledge and information. Biobanking, particularly biobanking with respect to human tissue samples, raises complex governance questions not only with respect to abundant biometric data but also with respect to individual privacy. It also requires careful attention to intersections between governance of shared genetic and related biological data, on the one hand, and preservation of physical samples themselves, which might degrade *via* overuse. Two different GKC-themed case studies, “Biobanks as Knowledge Institutions” (Madison, 2019) and “Population Biobanks' Governance: A Case Study of Knowledge Commons” (Boggio, 2017) explore those nuances.

### Genomics

Abstracted from their material context, genomic data pose few of the governance problems associated with tissue samples in biobanking. But the data generated by Human Genome Project and successors and alternatives expose the critical roles of information intermediaries in commons governance relative to massive quantities of information. Intermediary institutions ensure that data are organized and accessible for broad public use. Three GKC-themed case studies elaborate on that point, including “Leviathan in the Commons” (Contreras, 2017), “Genomic Data Commons” (Evans, 2017), and “Constructing the Genome Commons” (Contreras, 2014). The results illustrate the key point that knowledge commons governance is not necessarily opposed to integration with government-supplied resources of various sorts. Understanding polycentric governance requires seeing from all sides, not simply seeing like a state.

### Open Source Computer Programs

Open source computer software production is among the earliest areas of social practice drawing interest from researchers on community-based creativity and innovation. The success of the Linux project as the product of thousands of separate coders coordinated lightly *via* culture and a specific copyright license was a central part of Yochai Benkler's narrative in “The Wealth of Networks” (Benkler, 2006). But that work was pulled along by an ideological commitment to openness and to certain communitarian forms of social order. More nuanced, empirical work in Ostrom's footsteps describes the open source landscape in terms that emerged concurrently with the publication of the GKC framework (Schweik and English, 2012). The research did not hesitate to observe that the combination of abundant information and abundant programmers poses both substantial barriers to institutional success and opportunities for community-based innovation in institutional governance. A follow-up study, “Toward the comparison of open source commons institutions,” aligns that finding explicitly with the GKC framework (Schweik, 2014).

### Big Data

Last, “Tools for Data Governance” directly addresses governance problems associated with Big Data. It calls out the various dimensions of information abundance specifically as a justification for applying Ostromian thinking in general, as to institutional context and polycentricity, and the GKC research strategy outlined above, specifically (Madison, 2020). Unlike a lot of other work analyzing Big Data collection practices, this article does not prioritize privacy or surveillance considerations as first among all possibly relevant Big Data considerations, either normatively or descriptively.

## Conclusion

We've argued that contemporary rhetoric surrounding shifts from a scarcity-based society to an abundance-based society are partly underdone and partly overcooked. Underdone in that they fail to appreciate the long history of experience and analysis that focuses on various versions of abundance concepts and fail to appreciate the many ways in which abundance and scarcity are intertwined in practice. We're not seeing a massive shift from scarcity to abundance. We're seeing the emergence of contexts, some novel and some evolutionary, where the mix may be different and may be the same. And overcooked in that contemporary analysts often jump straight to celebrating the effects of abundance without looking carefully or critically at what's really happening on the ground.

We've built and used a framework to help researchers study the effects of abundance empirically, carefully, and systematically. It's the Governing Knowledge Commons research framework. It works. We have the cases, summarized here, to show that. It can be extended and improved. We hope that it will be *via* continuing research, across many diverse fields, through a wide variety of cases, and at macro, meso, and micro scales.

We don't imagine that the framework offers a theory of everything or answers all of the questions that abundance (or anything else) might pose. The framework generates only the data. It doesn't generate instant solutions. It doesn't yet do more than reinforce our initial intuition: that what's scarce and what's abundant are within our power largely to control, even if only imperfectly. Institutional design matters. Institutional design begins with contextual understanding.

## Data Availability Statement

The original contributions presented in the study are included in the article/supplementary material. Further inquiries can be directed to the corresponding author/s.

## Author Contributions

All authors listed have made a substantial, direct, and intellectual contribution to the work and approved it for publication.

## Conflict of Interest

The authors declare that the research was conducted in the absence of any commercial or financial relationships that could be construed as a potential conflict of interest.

## Publisher's Note

All claims expressed in this article are solely those of the authors and do not necessarily represent those of their affiliated organizations, or those of the publisher, the editors and the reviewers. Any product that may be evaluated in this article, or claim that may be made by its manufacturer, is not guaranteed or endorsed by the publisher.
